# NFAT1 Regulates Ly6C^hi^ Monocyte Recruitment to the CNS and Plays an Essential Role in Resistance to *Toxoplasma gondii* Infection

**DOI:** 10.3389/fimmu.2019.02105

**Published:** 2019-09-06

**Authors:** Luciana Benevides, Verônica M. Saltarelli, Franciele Pioto, Laís A. Sacramento, Murilo S. Dias, Gretel R. Rodríguez, João P. B. Viola, Vanessa Carregaro, João S. Silva

**Affiliations:** ^1^Department of Biochemistry and Immunology, Ribeirão Preto Medical School, University of São Paulo, Ribeirão Preto, Brazil; ^2^Fiocruz-Bi-Institutional Translational Medicine Plataform, Ribeirão Preto, Brazil; ^3^Program of Immunology and Tumor Biology, Brazilian National Cancer Institute (INCA), Rio de Janeiro, Brazil

**Keywords:** inflammatory monocyte, NFAT1, *T. gondii*, cell migration, Th1 cells

## Abstract

Monocytes play key roles in the maintenance of homeostasis and in the control of the infection. Monocytes are recruited from the bone marrow to inflammatory sites and are essential for antimicrobial activity to limit tissue damage and promote adaptive T cell responses. Here, we investigated the role of Nuclear Factor of Activated T cells 1 (NFAT1) in the regulation of Ly6C^hi^ inflammatory monocyte recruitment to the CNS upon *T. gondii* infection. We show that NFAT-1-deficient monocytes are unable to migrate to the CNS of *T. gondii*-infected mice. Moreover, NFAT1^−/−^ mice are highly susceptible to chronic *T. gondii* infection due to a failure to control parasite replication in the CNS. The inhibition of Ly6C^hi^ inflammatory monocyte recruitment to the CNS severely blocked CXCL10 production and consequently the migration of IFN-γ-producing CD4^+^ T cells. Moreover, the transfer of Ly6C^hi^ monocytes to infected NFAT1^−/−^ mice favored CD4^+^ T cell migration to the CNS and resulted in the inhibition of parasite replication and host defense. Together, these results demonstrated for the first time the contribution of NFAT1 to the regulation of Ly6C^hi^ monocyte recruitment to the CNS and to resistance during chronic *T. gondii* infection.

## Introduction

Monocytes are a subset of circulating white blood cells that can differentiate into tissue macrophages and dendritic cells in a CCR2-dependent manner (1). During homeostasis and inflammation, circulating monocytes leave the bloodstream and migrate into tissues following stimulation with cytokines, chemokines, and/or microbial products (2). Monocytes are crucial for the effective control and clearance of viral (3), bacterial (4), fungal (5), and protozoa (5–7) infections, but recruited monocytes also contribute to the pathogenesis of non-infectious inflammatory diseases (8).

Toxoplasmosis is a worldwide zoonotic infection caused by the opportunistic intracellular parasite *Toxoplasma gondii* (9). After oral infection, the parasites invade the intestinal epithelium and induce a strong Th1-type immune response associated with the production of IL-12 by dendritic cells (DCs), macrophages, and neutrophils, which drives the production of IFN-γ by T cells and NK cells, main mediator of acute defenses against the parasite (10). During the acute stage of *T. gondii* infection, monocytes produce high amounts of TNF-α, inducible NO synthase (iNOS), and reactive oxygen intermediates (ROS), which contribute to the control of the parasite burden in the host (11, 12). Upon acute infection, the parasites persist as cysts in the central nervous system (CNS) for the lifetime of the host. The control of cysts depends on the T-cell- and NK-cell-derived IFN-γ (13, 14). Moreover, subsets of myeloid cells, such as Ly6C^hi^CCR2^+^ inflammatory monocytes that infiltrate the CNS upon chronic *T. gondii* infection, play a decisive role in host defense, indicating that ongoing inflammation is required to control chronic cerebral infection (15).

Nuclear factor of activated T cells (NFAT) proteins constitute a family of transcription factors that includes the five members NFAT1 (also called NFATc2 or NFATp), NFAT2 (NFATc1 or NFATc), NFAT3 (NFATc4), NFAT4 (NFATx or NFATc3), and NFAT5 (TonE-BP or NFATL1) (16). The translocation of NFAT molecules from the cytoplasm into the nucleus induces the regulation of diverse cellular functions, such as proliferation, differentiation, and development (16). NFAT family proteins were first identified in T cells as activators of IL-2 transcription (17, 18), becoming key regulators of the T-cell immune response. Moreover, they are also involved in the control of the development and differentiation of T cells (19).

NFAT1, the first member of the NFAT family discovered in T cells, can be activated through dephosphorylation by calcineurin phosphatase. The dephosphorylation of NFAT1 controls the protein to be relocated into the nucleus and to be activated as a transcription factor (19). The NFAT1 pathway is clearly involved in T cell activation and, more recently, was shown to play a key role in innate cell activation. In macrophages, NFAT1 deficiency inhibits the production of pro-inflammatory cytokines, such as IL-12, IL-6, and TNF-α, even in response to LPS and ameliorates experimental colitis (20). NFAT1 signaling also controls the expression of genes related with DC apoptosis in response to LPS, an essential regulatory mechanism to restrain of the excessive immune activation (21). However, the role of NFAT1 signaling in inflammatory monocyte migration and the control of intracellular parasite infection is unknown.

Here, we evaluated the role of NFAT1 in the control of *T. gondii* infection. We found that the absence of NFAT1 leads to a high susceptibility to chronic *T. gondii* infection due to a failure to control parasite replication as a consequence of a deficiency in Ly6C^hi^ monocyte recruitment to the CNS. Moreover, we showed that NFAT1 contributes to Ly6C^hi^ monocyte migration and Th1 response to the CNS and confers resistance to chronic *T. gondii* infection.

## Materials and Methods

### Mice

NFAT1-deficient mice were generated on a mixed genetic 129Sv/J × C57BL/6 background as described (22). Eight-week-old female NFAT1^−/−^ mice and the littermate control (wild-type animals) were used in all experiments. Animals were bred and maintained in a specific pathogen-free animal facility at the University of São Paulo, Brazil and maintained in a pathogen-free environment. All procedures were performed in accordance with the International Guidelines for the Use of Animals and approved by the local Ethics Committee at the University of São Paulo, Brazil (CETEA/FMRP, protocol number 127/2011) and with standard biosecurity and Institutional safety procedures (CQB-0030/97).

### Infection

To the maintenance of the low-virulence ME-49 strain of *T. gondii*, C57BL/6 mice were infected with 10 cysts by intraperitoneal route and 30 days later the brains removed and mechanically homogenized in 3 ml of sterile PBS. The cyst numbers were determined using a light microscope. To the infection, the WT and NFAT1^−/−^ mice were orally infected by gavage with 100 *T. gondii* cysts in a total volume of 0.2 ml saline solution (23, 24). Survival was assessed daily (8 mice/group) for 50 days post-infection and at end of this time animals that survived or those exhibiting weight loss and with the signal of suffering were euthanized.

### Quantification of Tissue Parasitism by qPCR

The detection of parasite load was performed by the quantification of genomic parasite DNA. Briefly, genomic tissue DNA was extracted of 10 mg of tissue using the Wizard SV Genomic DNA Purification System Kit (Promega) and quantified with a spectrophotometer (Thermo Scientific NanoDropTM 1000). A sample of 100 ng of tissue from each animal was analyzed by quantitative PCR (qPCR) using GoTaq qPCR Master Mix fluorescence quantification systems (Promega). The reaction was performed in the StepOne Plus Real-Time PCR System (Applied Biosystem) using the following parameters: 2 min at 50°C; 2 min at 95°C; 14 cycles of 15 s at 95°C, 30 s at 58°C, and 30 s at 72°C; and a dissociation step with the temperature ranging from 60 to 95°C. Amplification of parasite DNA was performed with primers specific for the *T. gondii* B1 gene (sense: 5′-TTCAAGCAGCGTATTGTCGA-3′ and antisense: 5′-CATGAACGGATGCAGTTCCT-3′). The results were quantified based on the standard curve obtained by using the total genomic DNA extracted from *T. gondii* tachyzoites (ME-49 strain).

### Isolation of Leukocytes and Cell Culture

To characterize the inflammatory infiltrate in the CNS, the samples was collected and prepared as previously described (25, 26) with modifications. Briefly, for leukocytes isolation from the brains, the mice were anesthetized, perfused via intracardiac with the injection of 50 ml of PBS, followed by removal of the CNS. The whole brains were collected (*n* = 5 from each experimental group), minced in Hank's medium, and the remaining tissue was forced through a 70-mm cell strainer to obtain a single-cell suspension. The cell suspension was washed with PBS and fractionated by centrifugation for 45 min at 1,250 × g on a 30–70% Percoll gradient (GE Healthcare). The cells in the interphase comprised the mononuclear cells, which were harvested, washed with PBS, and viable cells determined using the trypan blue exclusion. Leukocytes from the CNS were stimulated with PMA (50 ng/ml) plus ionomycin (500 ng/ml) (Sigma) and brefeldin A (Biolegend) for the analysis of intracellular cytokines by flow cytometry. The spleens of mice were homogenized, and erythrocytes were lysed with 2 ml ACK lysis buffer (Thermo Fisher Scientific). The remaining cells were washed with PBS, and viable cells were counted via trypan blue exclusion. Single-cell suspensions from spleen were diluted to concentration 2 × 10^6^ cell/well, and dispensed into 48-well plates in a total volume of 500 μl of 5% fetal bovine serum in RPMI-1640 medium (Gibco, Life Technologies, USA) with or without soluble *T. gondii* antigen (STAg; 10 μg/ml). The STAg was prepared as previously described (27). Briefly, the ME-49 strain tachyzoites were cultured in LLCMK2 cell line, the parasites sonicated, centrifuged, the supernatant collected, aliquoted, and stored at −80°C until the use. As a positive control, we used anti-CD3 (2 μg/ml) plus anti-CD28 antibody (1 μg/ml) (BD Bioscience, USA). The minimal viability allowed was 95% and there was no significant variability between the experimental groups. The cell culture supernatants were harvested after 72 h of culture at 37°C in 5% CO_2_, and the levels of IFN-γ determined by ELISA with commercial Kits (R&D Systems, USA) according to the manufacturer's protocols.

### Flow Cytometry Assay

To staining the leukocytes, 1–2 × 10^6^ cells/tube were incubated with the antibodies diluted (100 times) during 30 min in PBS at 4°C. To intracellular staining, the cells were fixed with 4% paraformaldehyde for 11 min and permeabilized with 0.5% saponin for 30 min. After permeabilization, the antibodies were added and incubated for 30 min at 4°C. All antibodies used were obtained from BD Bioscience and Biolegend: CD3 (145-2C11), CD4 (RM4-5), CD8 (53-6.7), CD45 (16A), IFN-γ (XMG1.2), CD11b (M1/70), MHCII (M5/114.15.2), Ly6C (HK1.4), and CCR2 (SA203G11) conjugated to different fluorochromes (FITC, PE, APC, APC-CY7, PERCP, PE-Cy7, and Pacific Blue). Data acquisition and analysis were performed using the FACSCanto II and FlowJo software, respectively.

### Histological Analysis

The brains (*n* = 5 mice/group) were removed and fixed in 10% buffered formalin and paraffin processed. Tissue sections of 5-μm thickness were deparaffinized and stained with hematoxylin and eosin (H&E). Using a light microscope, the number of cysts was counted in 40 images of each animal of the group.

### Quantitative Real-Time PCR (qPCR)

RNA was extracted using an SV Total RNA Isolation System Kit (Promega) according to the manufacturer's instructions. cDNA was synthesized via reverse transcription (High Capacity Kit, Applied Biosystems). Real-time PCR for quantitative mRNA expression analyses was performed on a StepOne Plus Real-Time PCR System (Applied Biosystems) using a GoTaq qPCR Master Mix fluorescence quantification system (Promega) and the primers listed in Table S1. The standard PCR conditions were as follows: 50°C for 2 min; 95°C for 2 min; and 40 cycles of 15 s at 95°C, 30 s at 58°C, and 30 s at 72°C, followed by a standard denaturation curve. The mean threshold cycle (Ct) values were used to calculate the expression of the target gene, which was normalized to the housekeeping gene HPRT (hypoxanthine phosphoribosyltransferase) that allows for quantitative gene expression analysis using the formula 2^−ΔΔCt^.

### Adoptive Transfer of CD11b^+^Ly6C^hi^CCR2^+^ Monocytes

To obtain bone marrow cells, the animals were anesthetized with xylazine (12 mg/kg) and ketamine (115 mg/Kg) by ip route, euthanized by cervical dislocation, the femurs and tibiae isolated, the epiphyses removed, the bone flushed with a syringe filled with RPMI 1640, the bone marrow cells harvested and the cells homogenized (28). Bone marrow cells from mixed C57BL/6 and 129Sv/J background non-infected mice, and CD11b^+^Ly6C^hi^CCR2^+^ cells were sorted using BD FACS ARIA. Then, 8 × 10^5^ cells were injected intravenously into NFAT1^−/−^ mice (*n* = 3) 18 days post-infection. Twenty-two days later, the recipient mice were sacrificed, and the brains were removed, and the number of cysts and inflammatory scores were analyzed by H&E.

### Statistical Analysis

Statistical analysis was performed using an unpaired the Mann Whitney U Test (comparisons between two groups) and by ANOVA followed by Bonferroni's multiple comparison tests (the means of the different groups were compared). The Kaplan-Meier method was used to compare the survival rates of experimental groups. Mantel-Cox and chi-squared tests were used to compare the survival curves of the experimental groups. The significance of these parameters was calculated using a log-rank test (5.0 GraphPad Software). All values were considered significantly different at *P* < 0.05.

## Results

### NFAT1 Is Necessary to Control *T. gondii* Replication in the CNS

**Figure 1:**
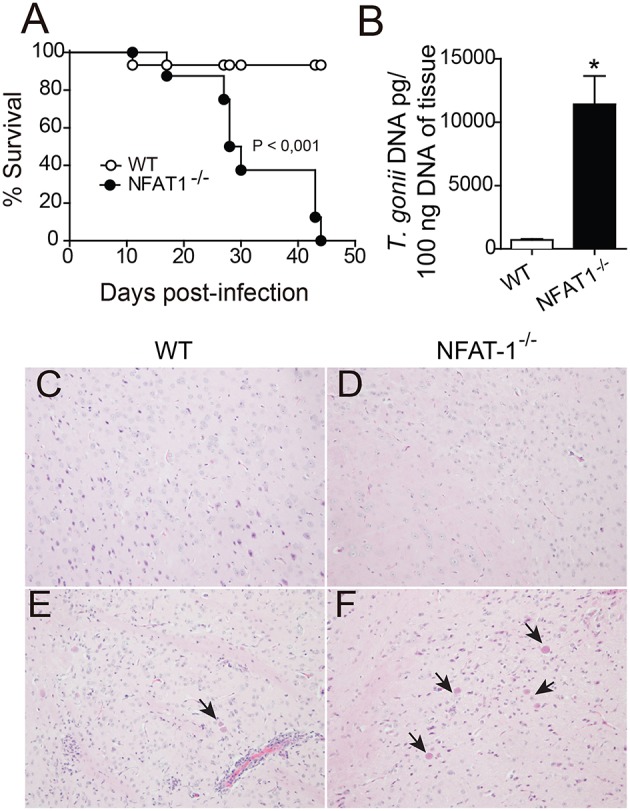
NFAT1 deficiency leads to an increased susceptibility to infection with *T. gondii*. WT and NFAT1^−/−^ mice were infected orally with 100 cysts from the ME-49 strain of *T. gondii*. Survival was evaluated daily **(A)**. Data are the mean ± SEM of 8 animals per experimental group of two independent experiments (*p < 0.001). At 25 days post-infection (dpi), the central nervous system (CNS) (*n* = 5 mice/group) was collected, the DNA of one hemisphere/mouse was extracted, and tissue parasitism was determined by qPCR based on a standard curve with *T. gondii* DNA **(B)**. Representative photomicrographs of CNS sections of WT **(C,E)** and NFAT1^−/−^ mice **(D,F)**, uninfected (top panel) and infected (bottom panel), stained with hematoxylin and eosin (H&E), are shown. Arrows indicate parasitophore vacuoles. Original magnification X200. Data are the mean ± SEM of the five mice per experimental group of two independent experiments (*P < 0.05).

To evaluate the role of NFAT1 on the resistance or susceptibility to infection by *T. gondii*, WT and NFAT1^−/−^ mice were infected orally with 100 cysts of the ME-49 strain *T. gondii*, and the survival was assessed daily. The data showed that NFAT1^−/−^ mice were highly susceptible to infection, with 100% of the animals succumbed by 45 dpi, while 90% of control animals (WT) survived up to 50 dpi (Figure 1A). At 25 dpi, we found that NFAT1^−/−^ mice harbored more parasites in the CNS than WT mice (Figure 1B). Histological analysis confirmed that NFAT1^−/−^ mice exhibited a higher number of parasitophorous vacuole in the brain when compared with that of WT mice (Figures 1C–F). We also found a higher parasite load in the liver and spleen of NFAT1^−/−^ mice compared to controls at 10 days post-infection (Figures S1A,B). In addition, in the absence of NFAT-1, the inflammatory infiltrate in the CNS was reduced compared to that of WT mice (Figures 1E,F). Together, these data indicate that NFAT1 contributes to the control of parasite replication in the CNS, suggesting that it is essential to the resistance to chronic infection by *T. gondii*.

### NFAT1 Plays an Essential Role in LY6C^hi^ Monocyte Recruitment to the CNS

**Figure 2:**
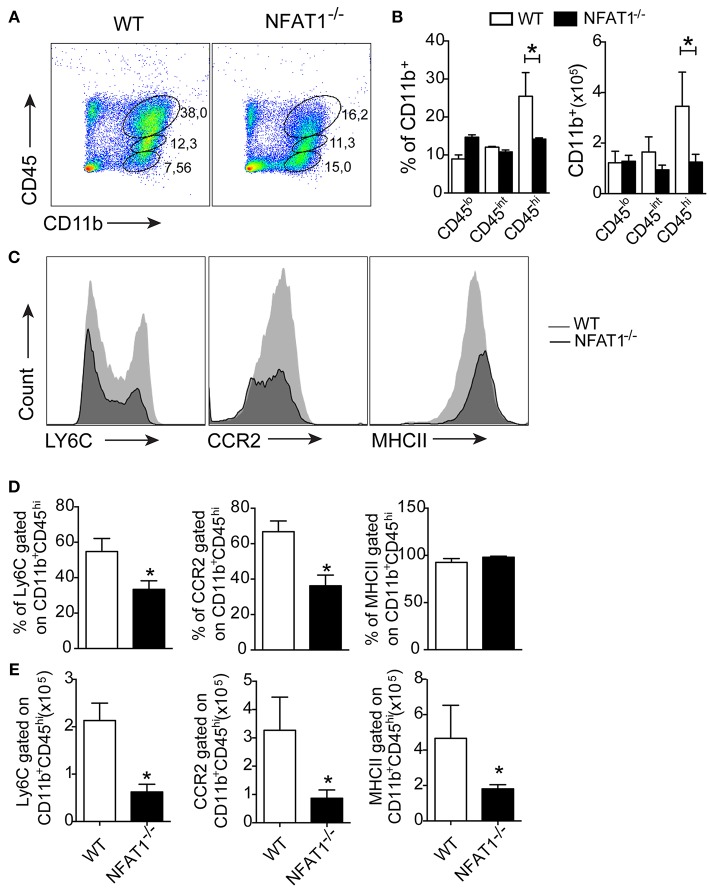
Deletion of NFAT1 results in failure of myeloid cell recruitment to the CNS. Mononuclear cells were isolated from the CNS of WT and NFAT1^−/−^ mice on day 25 post-infection with 100 cysts of the ME-49 strain of *T. gondii*. Dot plots **(A)** and bar graphs **(B)** show the percentage and the absolute number of myeloid (CD11b^+^CD45^hi^), activated microglia (CD11b^+^CD45^lo^) and resting microglia (CD11b^+^CD45^−^) populations obtained by flow cytometry. Representative histograms of the expression levels of LY6C, CCR2 and MHCII surface marker of the CD11b^+^CD45^hi^ population are shown **(C)**. Bar graphs show the percentage **(D)** and absolute number **(E)** of cells positive for LY6C, CCR2 and MHCII of the myeloid cells (CD11b^+^CD45^hi^) in the CNS from the WT and NFAT1^−/−^ mice at 25 dpi. Data are the mean ± SEM of the five mice per experimental group of two independent experiments (*P < 0.05).

Central toxoplasmosis is associated with the activation of resident cells and peripheral cell recruitment to the CNS (14). Here, we show that leucocytes (CD45^hi^) are in the brain in both mice lineages at 25 dpi (Figure 2). During the chronic phase of *T. gondii* infection, the frequency and absolute numbers of resting (CD45^−^CD11b^+^) and activated (CD45^int^CD11b^+^) microglia were similar in NFAT1^−/−^ and WT mice (Figures 2A,B). However, we found a reduction in the frequency and absolute numbers of inflammatory leukocytes (CD45^hi^CD11b^+^) in the CNS of NFAT1^−/−^ mice compared to WT mice (Figures 2A,B). Next, we characterized mononuclear cell subsets upon cerebral *T. gondii* infection. We first compared the expression of specific surface molecules of myeloid cells. No difference was found in CD45^int^CD11b^+^ cells in either lineage of mice. However, we found a reduction in the frequency and absolute number of LY6C^+^ and CCR2^+^ cells gated on CD11b^+^CD45^hi^ cells in the CNS from NFAT-1^−/−^ mice compared to WT mice (Figures 2C–E). However, despite the similar frequency of MHCII^+^ cells between both groups, we found a reduction of MHCII in CD11b^+^CD45^hi^ cells from NFAT1^−/−^ mice compared to WT mice (Figures 2C–E). Interestingly, NFAT1 deficiency did not alter the frequency and the absolute numbers of myeloid cells—CD11b^+^ (Figures S2A,B) and inflammatory monocytes (CD11b^+^LY6C^hi^) (Figures S2C,D) or their activation status in the peripheral tissues (Figures S2E,F) in the bone marrow and spleen of *T. gondii* infected mice. In addition, similar data were obtained between uninfected WT and NFAT1^−/−^ mice.

**Figure 3:**
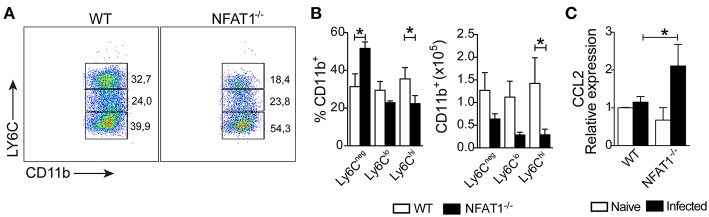
Lack of NFAT1 inhibits LY6C^hi^ monocyte migration to CNS. The mononuclear cells were isolated from the CNS of WT and NFAT1^−/−^ mice at day 25 post-infection with 100 cysts of the ME-49 strain of *T. gondii*. The myeloid cells (CD11b^+^CD45^hi^) were analyzed by the expression of Ly6C^hi^, LY6C^lo^, and Ly6C^neg^ by flow cytometry **(A)**. The frequency and the absolute number of the monocyte subsets are shown **(B)**. The relative expression of CCL2 mRNA in the CNS from WT and NFAT1^−/−^ mice was evaluated at 25 dpi by qPCR **(C)**. The data shown are representative of the mean ± SEM of five mice per experimental group of two independent experiments (*P < 0.05).

Next, we asked whether the migration of monocytes into the CNS was affected. We found a reduction in the frequency and absolute numbers of Ly6C^hi^ inflammatory monocytes gated on CD11b^+^CD45^hi^ in the CNS from NFAT1^−/−^ mice compared to WT mice (Figures 3A,B). Although *T. gondii* infection induces the migration of Ly6C^lo^ and Ly6C^neg^ monocytes gated on CD11b^+^CD45^hi^ in the CNS, the frequency and absolute number were similar in both experimental groups (Figure 3B). Because monocyte recruitment is due to CCL2 production, we examined its expression in the CNS during the chronic phase of infection. We found that in the absence of NFAT1, the expression of CCL2 was increased in the CNS compared to that of WT mice (Figure 3C), suggesting that the reduction of Ly6C^hi^ monocyte migration was not due to the deficiency in CCL2 expression. Together, our data showed that NFAT1 is essential for monocyte recruitment to the CNS during *T. gondii* infection.

### Deficiency of NFAT1 Results in Inhibition of Th1 Cell Migration to the CNS During *T. gondii* Infection

**Figure 4:**
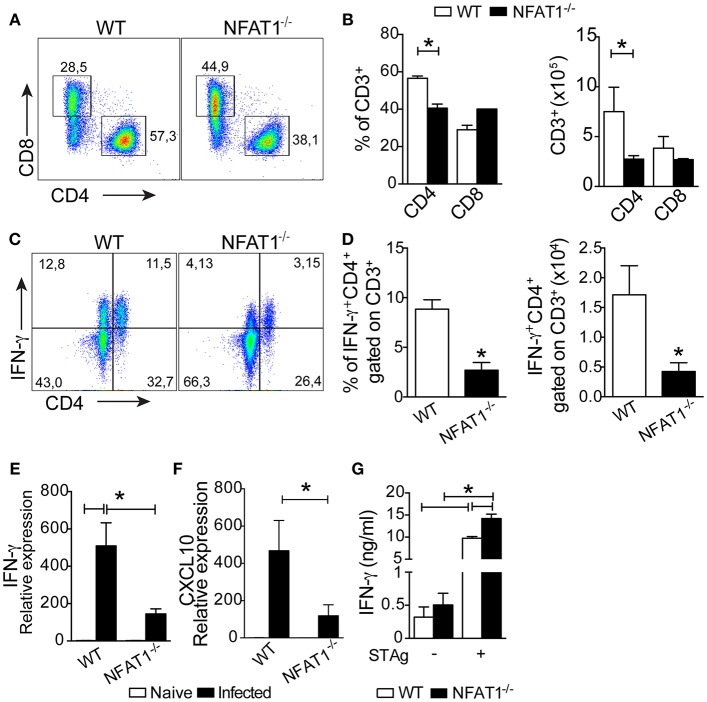
NFAT1 controls the recruitment of CD4^+^ T cells to the CNS. Mononuclear cells from the CNS of WT and NFAT1^−/−^ mice at 25 dpi with *T. gondii* were stained for CD4 and CD8. Dot plots **(A)** and graph bars **(B)** show the frequency and absolute number of CD4^+^ and CD8^+^ cells gated on CD3^+^ cells obtained by flow cytometry. The percentages and absolute numbers of IFN-γ-expressing CD4^+^ cells gated on CD3 were determined by flow cytometry in mononuclear cells isolated from the CNS that were stimulated with phorbol 12-myristate 13-acetate plus ionomycin for 4 h for intracellular staining **(C,D)**. The relative expression of IFN-γ **(E)** and CXCL10 **(F)**. The levels of IFN-γ were measured in the culture supernatants of splenocytes from WT and NFAT1^−/−^ mice infected with *T. gondii* and stimulated with *T. gondii* tachyzoites *soluble* antigen (STAg) for 72 h **(G)**. Data are expressed as the mean ± SEM of five mice per experimental group of two independent experiments (*P < 0.05).

To evaluate whether the deletion of NFAT1 compromises the migration of T lymphocytes to the CNS during *T. gondii* infection, we quantified the frequency and absolute numbers of CD4^+^ and CD8^+^ lymphocytes by flow cytometric analysis. At 25 dpi, we found a reduction in the frequency and absolute number of CD4^+^ T cells, but not CD8^+^ T cells, in the CNS of NFAT1^−/−^ mice compared to WT mice (Figures 4A,B).

In addition, together with the reduced number of CD4^+^ T cells, the frequency and number of CNS CD4^+^ T cells producing IFN-γ was smaller in NFAT1^−/−^ mice than in WT mice (Figures 4C,D). Consistently, the expression of IFN-γ (Figure 4E) and CXCL10 mRNA (Figure 4F) were drastically reduced in the CNS of NFAT1^−/−^ mice compared to WT mice at 25 dpi. In contrast, we found that splenocytes from NFAT1^−/−^ mice infected and stimulated with STAg for 72 h produced higher levels of IFN-γ compared to WT mice (Figure 4G). These data suggest that the migration of Th1 cells to the CNS during the chronic phase of *T. gondii* infection is dependent on NFAT1.

### Adoptive Transfer of Ly6C^hi^ Monocytes Controls Cerebral Toxoplasmosis in NFAT1-Deficient Mice

**Figure 5:**
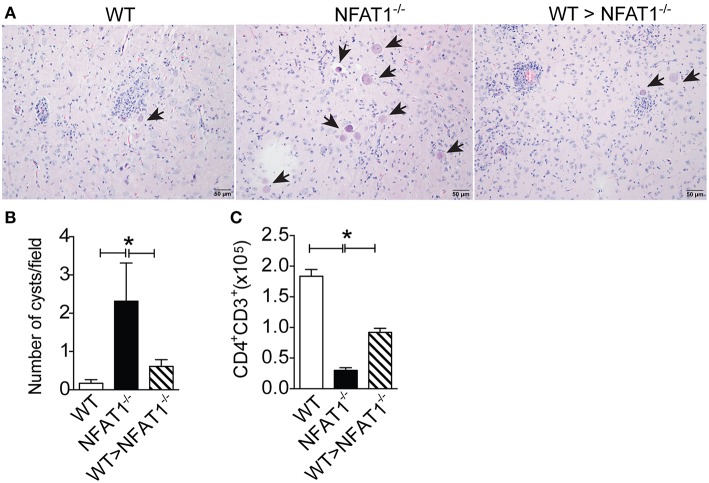
NFAT1 expression on Ly6C^hi^ monocytes plays an essential role in the resistance to *T. gondii* infection. Sorted CD11b^+^LY6C^hi^CCR2^+^ monocytes from WT mice bone marrow were injected i.v. into NFAT1^−/−^ mice 18 dpi with *T. gondii*. Twenty-two days later, the number of cysts and the inflammation score of the recipient mice were analyzed in tissue samples stained with H&E **(A,B)**. Arrows indicate parasitophore vacuoles. Original magnification X200. The mononuclear cells of WT and NFAT1 recipient mice were isolated from the CNS, and the absolute number of CD4^+^-gated CD3 cells was determined by flow cytometry **(C)**. Data are expressed as the mean ± SEM of three mice per experimental group of the two independent experiments (*P < 0.05).

Finally, we asked whether the failure of parasite replication control was due the deficiency of Ly6C^hi^ monocyte recruitment to the CNS in NFAT1^−/−^ mice. Therefore, we adoptively transferred CD11b^+^Ly6C^hi^CCR2^+^ cells isolated from the bone marrow of WT mice i.v. to *T. gondii*-infected NFAT1^−/−^ recipient mice at day 18 post-infection. Twenty-two days later, we found a reduction in the number of cysts in the CNS of the recipient NFAT1^−/−^ mice when compared to the infected NFAT1^−/−^ mice (Figures 5A,B). In addition, the transfer of Ly6C^hi^ monocytes to NFAT1^−/−^ mice resulted in increased numbers of CD4^+^ T cells in the CNS (Figure 5C). These data showed that the recruitment of Ly6C^hi^ monocytes to the CNS is essential to control parasite replication in a CD4^+^ cell-dependent manner.

## Discussion

Here, we demonstrated that NFAT1 controls the recruitment of LY6C^hi^CCR2^+^ monocytes to the CNS and is therefore essential for resistance to *T. gondii* infection. Upon cerebral inflammation, leukocyte recruitment from blood through blood-brain barrier is dependent on adhesion molecules and their receptors (29). The egress of the inflammatory monocytes from bone marrow occurs through CCR2-CCL2 axis, however the mechanism of crossing the blood barrier is poorly understood (30). Here, we showed that the mechanism of monocyte migration to the CNS takes place. In fact, the deficiency in NFAT1 compromised the recruitment of LY6C^hi^ inflammatory monocytes to the CNS during *T. gondii* infection, making the NFAT1-deficient mice unable to control the parasites, resulting in its death. It is important to note that the deletion of NFAT-1 did not affect the number of myeloid cells and inflammatory monocytes and their activation state in peripheral tissues, even during infection with *T. gondii*.

It is clear that NFAT1 plays a role in T cell homeostasis, but its involvement in monocyte migration and function is unclear. Consistent with previous studies, NFAT4 protein is involved in CCL2 and CXCL2 induction and neutrophil recruitment, acute pancreatic model (31) and *T. gondii* infection (32). NFAT1 is associated with a wide range of tumor progression events, such as invasion, migration, tumor cell survival and apoptosis, as shown in esophageal squamous cell carcinoma (33) and melanoma models (34). Our data, differently, show the role of NFAT1 in the migration of inflammatory monocytes to the CNS in *T. gondii* infected mice. Therefore, the failure of the monocytes recruitment to CNS in NFAT-1^−/−^ mice was not due to the lack of CCL2 chemokines. Regarding the NFAT1-induced resistance to infection, we showed that NFAT1 is necessary to control *T. gondii* replication in the acute and chronic phase of infection. Indeed, the control of parasite in the CNS was dependent of NFAT^+^ monocytes, because transference of Ly6C^hi^ monocytes from WT mice on 18 days post-infection, when the parasites is already in the CNS, controlled cerebral toxoplasmosis (decreased the number of cysts and increased the CD4^+^ T cells). Thus, we showed the crucial role of NFAT-1 in the inflammatory monocytes recruitment to CNS and to resistance to infection.

Overall, monocytes may have beneficial or detrimental distinct roles to the host during infections. In *Listeria monocytogenes* infection, the absence of Ly6C^hi^ monocytes leads to rapid death of mice, indicating their essential role in host defense (35, 36). Furthermore, in viral infections, Ly6C^hi^ monocytes infiltrate the CNS and, although contributing to viral clearance (3), favor immunopathology (37, 38). In intracellular parasite infections such as *T. gondii* (11, 39) and *Leishmania* sp. (40, 41) Ly6C^hi^ inflammatory monocytes are recruited to acute infection sites and control parasite replication.

In contrast, Ly6C^hi^ inflammatory monocytes are detrimental to the host and enhance pathology during *Trypanosoma brucei* infection. CCR2-deficient mice are more resistant to infection due to better control of the circulating parasite (42). The depletion of these cells prevents immunopathology and favors resistance to infection (43).

The infiltrating inflammatory monocytes at the site of infection is essential to the development of an adaptive immune Th1 protective response during protozoan infection (40). In the chronic phase of *T. gondii* infection, parasites persist in cysts within immune-privileged sites (44). The latent stage is associated with marginal inflammation, leukocytes migration to the CNS, which is crucial to production of IFN-γ that activates microglia cells to eliminate parasites (45, 46). Our data showed that the deficiency in NFAT1 did not change the numbers or activation of microglia during *T. gondii* infection. However, the lack of NFAT1 signaling is associated with the reduction of CXCL10 secretion and IFN-γ-producing CD4^+^ T lymphocyte migration to the CNS during *T. gondii* infection. The deficiency of NFAT1 compromises the infiltration of inflammatory monocytes into the CNS and consequently CXCL10 production, a chemokine known to be produced by monocytes and involved in T cell migration (47). The reduction of CXCL10 results in decreased migration of CD4^+^ T lymphocytes to the CNS and the production of IFN-γ in NFAT1^−/−^ mice. Although we found no difference in the amount of CD8^+^ T cells, the frequency IFN-γ producing these cells was reduced compared to that found in WT mice. In fact, we cannot exclude the participation of CD8 cells in this context, since both CD4 and CD8 T lymphocytes are important for the maintenance of resistance in the chronic phase of *T. gondii* infection (48, 49). Although NFAT1 signaling is related to the activation of T lymphocytes (50) and Th1 differentiation (51), we showed that the absence of NFAT1 did not change the capacity of T cell activation or Th1 cell differentiation. It is clear because the culture of splenocytes from NFAT1^−/−^ mice stimulated with STAg produced high levels of IFN-γ compared to WT mice. There is a defect in the migration of CD4^+^ lymphocytes to the CNS and consequently the production of IFN-γ, suggesting that this migration deficiency in NFAT1^−/−^ mice was due to the reduction in inflammatory monocyte recruitment to the CNS. Indeed, transfer of monocytes to the NFAT1^−/−^ mice partially control of parasites and restored the CD4^+^ T lymphocyte recruitment to the CNS and longer survival up to 40 days post-infection. Although survival was not followed for longer time, these data suggest that monocyte transferred to NFAT1^−/−^ mice provided a global benefit, favoring the resistance to infection. In conclusion, our findings indicate that in the absence of NFAT1, there is a failure to control parasite replication in the CNS due to inhibition in the monocyte Ly6C^hi^ recruitment to the CNS, which consequently compromises CD4^+^ T lymphocyte migration to and IFN-γ production at the infection site during the chronic stage. Furthermore, we showed that the adoptive transfer of Ly6C^hi^ monocytes to NFAT1^−/−^ mice induces an increase in migration of CD4^+^ T cells to the CNS and controls cerebral toxoplasmosis. Thus, our data suggest that NFAT1 signaling activation is essential for the migration of monocytes and, consequently, a Th1 cell response to the CNS, which is crucial for resistance to *T. gondii* infection.

## Data Availability

All datasets generated for this study are included in the manuscript and/or the Supplementary Files.

## Ethics Statement

All procedures were performed in accordance with the International Guidelines for the Use of Animals and approved by the local Ethics Committee at the University of São Paulo, Brazil.

## Author Contributions

LB and JS conceived and designed the experiments. LB, VS, FP, LS, MD, and GR performed the experiments. LB and JS analyzed the data. LB, JV, VC, and JS contributed reagents, materials, analysis tools. LB and JS wrote the paper.

### Conflict of Interest Statement

The authors declare that the research was conducted in the absence of any commercial or financial relationships that could be construed as a potential conflict of interest.
