# Supplementary material for: NFAT1 Regulates Ly6Chi Monocyte Recruitment to the CNS and Plays an Essential Role in Resistance to Toxoplasma gondii Infection
Source: Front Immunol. 2019 Sep 6;10:2105. doi: 10.3389/fimmu.2019.02105 (PMC6742953; doi:10.3389/fimmu.2019.02105)
Supplement: Supplementary file 1 [file Data_Sheet_1.doc]

**Supplementary Table S1. Murine primer sequences used in the study**

| **Gene** | **Foward (5’-3’)** | **Reverse (5’-3’)** |
| --- | --- | --- |
| CCL2 | AGAAGGAATGGGTCCAGACA | TCATTTGGTTCCGATCCAG |
| IFN-γ | CATGGCTGTTTCTGGCTGTTAC | CCAGTTCCTCCAGATATCCAAGA |
| CXCL10 | AGCGTTTAGCCAAAAAAGGTC | TGGCTTCACTCCAGTTAAGGA |
| HPRT | AACAAAGTCTGGCCTGTATCC | CCCCAAAATGGTTAAGGTTGC |


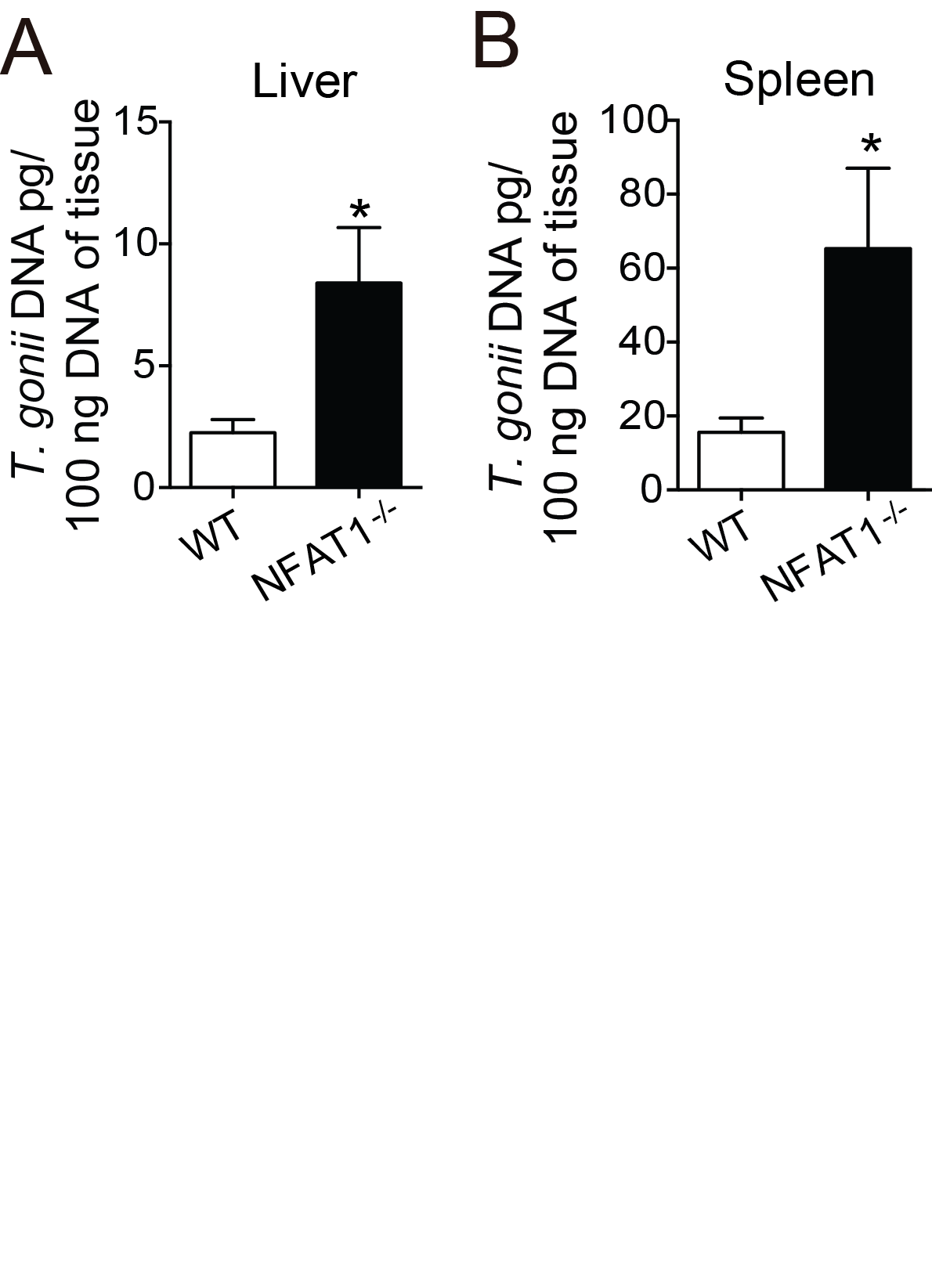


**Supplementary Figure S1. NFAT1 controls tissue parasitism in the acute phase of *T. gondii* infection.** WT and NFAT1^-/-^ mice were infected orally with 100 cysts from the ME-49 strain of *T. gondii*. At 10 days post-infection (dpi), the liver **(A)** and spleen **(B)** were collected and tissue parasitism was determined by qPCR based on a standard curve with *T. gondii* DNA. Data are the mean ± SEM of the four mice per experimental group (* P < 0.05).

**
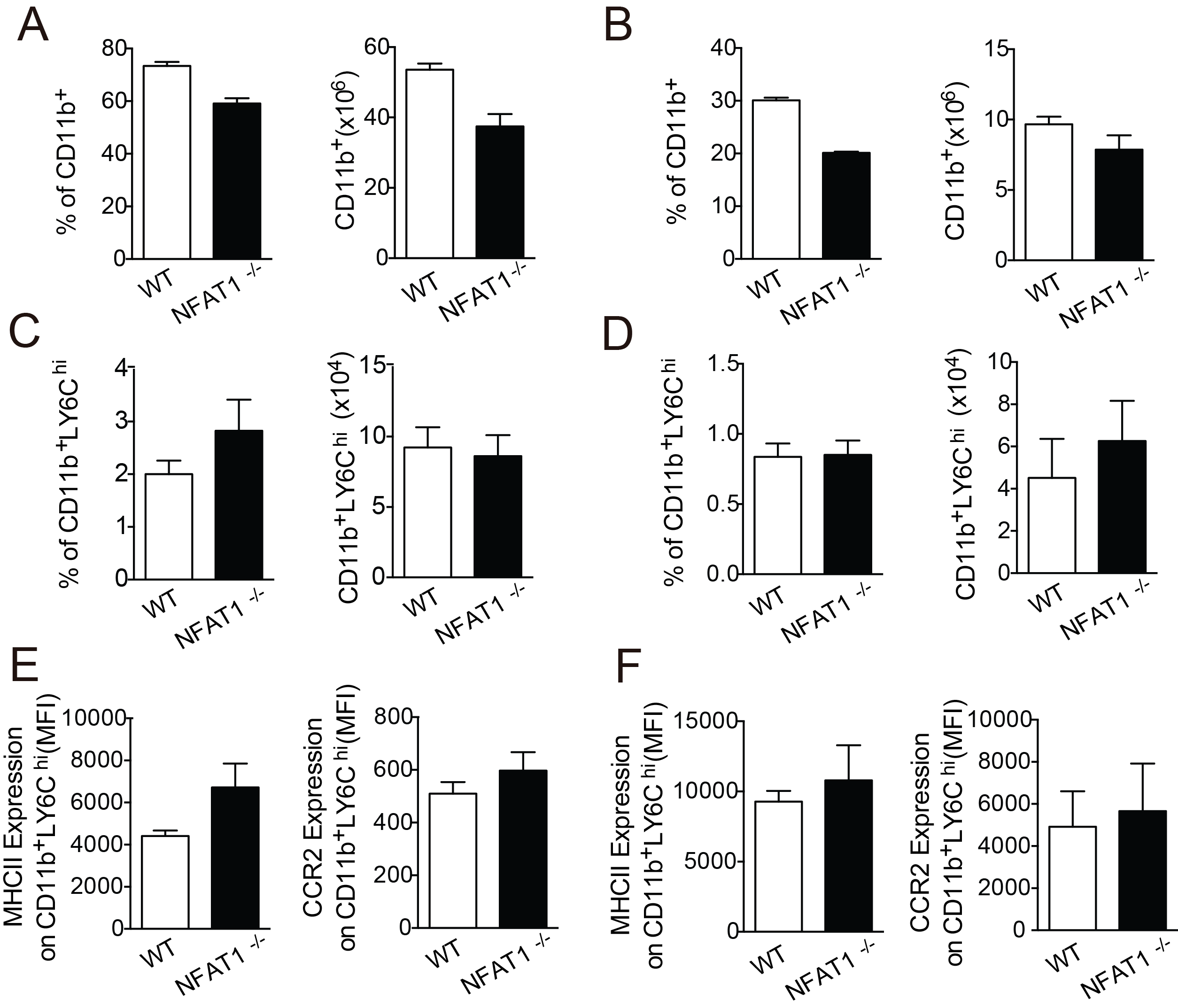
**

**Supplementary Figure S2. NFAT1 deficiency did not alter the number of monocytes and their activation state in peripheral tissues during *T. gondii* infection.** The bone marrow cells **(A, C and E)** and spleen cells **(B, D and F)** were isolated of WT and NFAT1^-/-^ mice at day 25 post-infection with 100 cysts of the ME-49 strain of *T. gondii*. The frequency and absolute numbers of myeloid cells (CD11b^+^) were analysed by flow cytometry **(A and B)**. The frequency and the absolute number of the inflammatory monocyte (CD11b^+^LY6C^hi^) cells are shown **(C and D)**. Expression of MHCII and CCR2 on surface CD11b^+^LY6C^hi^ cells were analysed by mean fluorescence intensity, MFI by flow cytometry **(E and F)**. The data shown are representative of the mean ± SEM of four mice per experimental group of two independent experiments (* P < 0.05).
